# Comparison of vaccine hesitancy during the low and high points of COVID-19 in a population under international sanctions: A longitudinal mixed-methods study in Iran

**DOI:** 10.3389/fpubh.2022.958899

**Published:** 2023-01-05

**Authors:** Hamidreza Khankeh, Mohammad Pourebrahimi, Mohammadjavad Hosseinabadi-Farahani, Mehrdad Farrokhi, Mohammad Saeed Khanjani, Javad Shojafard, Arya Hamedanchi, Maryam Ranjbar, Mariye Jenabi Ghods, Shokoufeh Ahmadi, Elham Ghanaatpisheh, Mohammad Saatchi

**Affiliations:** ^1^Health in Emergency and Disaster Research Center, University of Social Welfare and Rehabilitation Sciences, Tehran, Iran; ^2^Department of Clinical Science and Education, Karolinska Institute, Stockholm, Sweden; ^3^Nursing Faculty, Baqiyatallah University of Medical Sciences, Tehran, Iran; ^4^Department of Nursing, University of Social Welfare and Rehabilitation Sciences, Tehran, Iran; ^5^Department of Counseling, University of Social Welfare and Rehabilitation Sciences, Tehran, Iran; ^6^Iranian Research Center on Aging, University of Social Welfare and Rehabilitation Sciences, Tehran, Iran; ^7^Master of Psychiatric Nursing, University of Social Welfare and Rehabilitation Sciences, Tehran, Iran

**Keywords:** SARS-CoV-2, vaccination hesitancy, vaccine hesitancy, COVID-19 vaccines, Iran

## Abstract

**Introduction:**

Along with the challenges of COVID-19 vaccine supply in low-income countries, vaccine hesitancy was another problem for the health system. The aim of this study was to deeply understand the challenges of vaccine acceptance, the vaccination process, and to compare the affecting vaccine acceptance in the high and low points of the epidemic in Iran.

**Methods:**

In the qualitative part of this mixed-methods study, content analysis was used to investigate experiences and perceptions about COVID-19 vaccination in four groups. In the quantitative study, in March 2021 (low point), and on August 1, 2021 (high point), two population-based cross-sectional studies were performed in Tehran and its rural, with sample sizes of 1,200 and 1,872 people aged over 18 years, respectively. Multinomial (polytomous) logistic regression was used to determine the factors affecting hesitation and unwillingness to receive the vaccine.

**Results:**

Disbelief in vaccine safety, vaccine distrust, ignorance and confusion, and inadequate facilities were the common reasons extracted in the two qualitative studies. At the low and high points of the epidemic, vaccine acceptance was 83.6% (95% CI: 81.3–85.9) and 65.8% (95% CI: 65.8–71.0), respectively. Residence in rural areas, (Odds Ratio: 0.44, *p* = 0.001), being a student (Odds Ratio: 0.41, *p* = 0.011), housewives (Odds Ratio: 0.63, *p* = 0.033), illiteracy (Odds Ratio: 4.44, *p* = 0.001), and having an underlying disease (Odds Ratio: 4.44, *p* = 0.001) were factors affecting on vaccine acceptance.

**Discussion:**

Counter-intuitively, acceptance did not increase at the peak of epidemic. The presence of obstacles, such as increased distrust in the effectiveness of vaccines due to the occurrence of multiple peaks in different vaccinated countries, as well as the influence of the media, anti-vaccine campaigns, and lack of proper communication about risks caused more hesitation. More investigation to understand how people accept or reject vaccine and its long term consequences is recommended.

## Introduction

Although the Iranian health system is one of the most developed in the Eastern Mediterranean Region (1), unfortunately, due to the complexity, contagiousness, and unpredictability of COVID-19, Iran, like many countries, has faced many waves of the disease (2); as such, vaccination, alongside public health recommendations, was seen as crucial to preventing its spread (3). Although developed countries started the vaccination of the elderly and Health Care Workers (HCW) and even parts of the general population despite the lack of full approval of the vaccines, in Iran, this process started later due to the continuing study phases of the vaccines, insufficient access to international vaccines due to political sanctions, and disagreement among experts regarding the available vaccines due to their emergency approval process. In addition to the challenges of vaccine supply, opponents of the vaccine also spread misinformation about the ineffectiveness of the vaccine in preventing COVID-19 and its numerous side effects, such as infertility and death, through the Internet and in both local and scientific communities; as a result, health officials became concerned about increasing skepticism of the vaccine. According to the World Health Organization, vaccine hesitancy was one of the top ten global health threats in 2019 (4), and with the epidemic of the COVID-19, it became important to clearly identify the roots and causes of vaccine hesitancy. Although many researchers in different parts of the world have studied the reasons for unwillingness to take the COVID-19 vaccine (5–10), it seems that the performance of national health systems, trust and social capital, economic status, and availability of transparent information about the vaccine play a decisive role in doubts about receiving the vaccine. On the other hand, changing risk perceptions across low and high waves of the epidemic is another indicator that is expected to play a role in the acceptance of the COVID-19 vaccine. International sanctions, confrontations between anti-vaccine campaigns and the scientific community in Iran's unique socio-cultural context, and the emergence of epidemic waves make COVID-19 vaccine acceptance a special case. COVID-19 vaccine hesitancy is a complex phenomenon, and it is not possible to understand people's experiences and perceptions about the reasons for hesitancy by conducting a quantitative study alone. Using qualitative studies with quantitative parts could offer explanations for unexpected findings generated by quantitative. In general, researchers were interested to know how people experience vaccination and vaccine hesitancy, the meaning of this experience, and the context in which this experience is embedded. For the sake of clarity of situation of vaccine hesitancy in the context of Iranian culture, the combination of qualitative and quantitative study results would be an acceptable strategy for evidence-based decision making. Therefore, the aim of this study was to deeply understand the obstacles to vaccine acceptance and the vaccination process and to determine the factors affecting vaccine acceptance in Tehran and its rural areas in the high point of the COVID-19 epidemic and also in the following low point of the COVID-19 epidemic.

## Materials and methods

The present study was a concurrent mixed-methods study that was conducted in Tehran and its surrounding rural areas. Mixed-methods research emphasizes collecting, analyzing, and combining quantitative and qualitative data in a single study (11, 12). Given that both qualitative and quantitative approaches have weaknesses and limitations in methodology and presentation of results, mixed-methods studies can compensate for these shortcomings. Thus, the use of an integrated approach is motivated by the research question and offers a wider range of methodological perspectives, increases the overall validity of the results, and brings the researcher closer to the recognition of reality (13).

### Qualitative section

The two qualitative phases of the study analyzed participants' experiences and perceptions of COVID-19 vaccination in low and high points using the content analysis method. Participants in the both studies were selected purposefully for maximum diversity, and sampling was continued until data saturation was achieved. Inclusion criteria included being a Health Care Worker (HCW), elderly, people, or having an underlying disease (cardiopulmonary disease, cancer, diabetes, hemodialysis, autoimmunity, and transplant history); in the general population, inclusion criteria were being aged over 18 years, verbal ability to participate in the interview, and willingness to participate in the study. Data collection was performed during the low point of COVID-19 (April and May 2021) and the high point of COVID-19 (June and July 2021) using in-depth, semi-structured interviews with open-ended questions. Preliminary interviews were conducted by the first author, who is an expert in interviewing and conducting qualitative studies.

Interviews were started with a brief description of the participant's personal characteristics and then the question “How did you experience the COVID-19 vaccination process in Iran?” Then, according to the purpose and methodology of the study, the questions were asked with the aim of identifying the hidden and deep layers of the participants' experiences and perceptions. Probing questions, such as “What made you make this decision?”, “What do you mean?”, “Please explain more?”, “How?”, and “What was your reaction to these events and information?” were also asked. The interviews continued until the participants believed that there was nothing left to say, and, finally, they were ended with the question “Does anything else come to your mind that you want to say?” and “If something comes to mind, you can call me.” In addition to interviews, data was also collected by observing and reviewing documents. The research team simultaneously analyzed the experiences and perceptions of the study participants about the vaccination. In this study, data analysis was performed using the method of Graneheim and Lundman.

To increase the trustworthiness, the four criteria of Lincoln and Guba were used to ensure the credibility of the results: member check, expert check, and peer check. For this purpose, all the interviews were coded separately by two researchers, and disagreements were resolved in meetings. Validation was performed during the interview by restating or summarizing information and asking the participant to verify. Also, specially coded interviews were reviewed by participants to ensure researchers' interpretations of the data were reasonable. For external audit, the data were reviewed by an independent expert with extensive experience in qualitative research. To confirm the results, the research processes are described in detail to make it possible to follow up the research. To improve the transferability of the results, the demographic characteristics of the participants and the topic of interest are described in detail to allow the reader to decide how to interpret the results.

### Quantitative section

#### Low point phase

The study design of the first study has already been published (14). In summary, in March 2021, before the start of the fourth wave of the epidemic in Iran, a population-based cross-sectional study was conducted in Tehran, with a sample size of 1,200 individuals aged more than 18 years. To determine COVID-19 vaccine acceptance and the factors affecting it, a questionnaire designed by researchers was used, which was administered by going to the participants' houses and interviewing them.

#### High point phase

##### Study design

**Figure 1:**
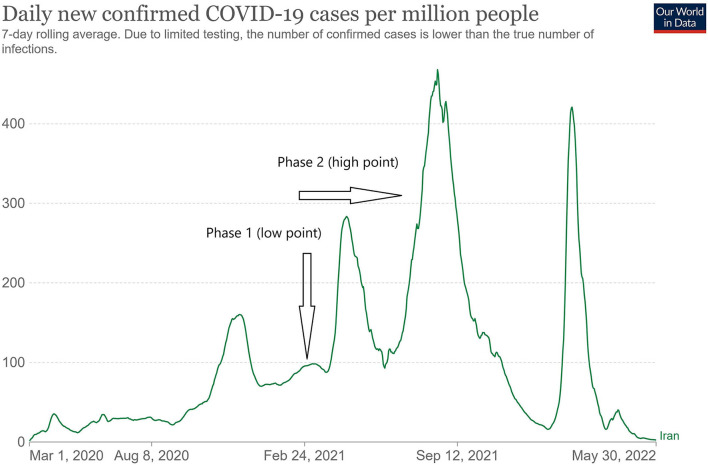
The trend of the number of daily new COVID-19 cases in Iran (15) and the time of implementation of two studies on vaccine acceptance in Tehran and its rural areas at the low and high points of the epidemic.

From late June 2021, the fifth wave of the COVID-19 epidemic occurred in Iran, as daily confirmed new cases sharply increased [Figure 1, (15)], and on August 1, 2021, the second phase of the vaccine acceptance study began in 22 districts of Tehran and 10 selected rural areas as a population-based cross-sectional study. Inclusion criteria included living in Tehran or rural areas and the age of 18 years and older, and eligible individuals who did not want to participate in the study were excluded. The multi-stage cluster sampling method was used for sampling from households in 22 districts of Tehran. The required sample size in this survey was estimated to be 1,400 people in urban areas and 400 people in rural areas, for a total of 1,800 people, according to the type of sampling and design effect. The neighborhoods of the 22 districts were the clusters, and after randomly selecting the clusters, at least 30 households from each cluster were selected and one of them was interviewed.

##### Study tools

A researcher-made questionnaire was used to collect the data. The questions on this questionnaire were revised using the results of the first phase of the study on vaccine acceptance in Tehran by the same research team and also by reviewing the content, using the experiences of research team members, and evaluating the face and content validity. This questionnaire consisted of 42 questions in 5 subgroups. The sections and related variables of this questionnaire are provided in Supplementary Table 1. Regarding participants' health status, the answers to the self-rated health question were reported on a Likert scale from “very good” to “very bad.” However, this independent variable was re-grouped as “good,” “moderate,” and “bad” in the logistic regression model. Based on experiences from the first study, changes were made to the third part of the questionnaire (information about receiving the vaccine and the willingness to receive the vaccine). Participants were first asked whether or not they had received at least one dose of the vaccine by the time of the present study. If they hadn't, respondents were asked, “If the vaccine is approved by the Ministry of Health of Iran (MOH), will you apply for the vaccine?” and it was answered using three options: “Yes” (vaccine acceptance), “No” (vaccine hesitancy), and “Unsure.” In the first study, however, there were only “Yes” and “No” options.

### Statistical analysis

Mean ± SD and percentages were used to report quantitative and classified variables, respectively. The prevalence of “Yes,” “No,” and “Unsure” responses was calculated and is reported with 95% confidence intervals for participants who had not received the vaccine by the time of the study in general and separately for urban and rural areas. To determine the factors affecting the “Unsure” and “No” responses, multinomial (polytomous) logistic regression was used. In this method, the “Yes” group was used as the basis and the exponential coefficients estimated from the model reported as unadjusted and adjusted Relative-Risk Ratio of “Unsure” and “No” responses. All analyses were performed using stata14.0 software (StataCorp LLC, 4905 Lakeway Drive College Station, TX USA) at an error level of 5%.

## Results

### Qualitative section

We had 45 and 42 participants for the low and high points, respectively. The characteristics of the participants in the qualitative part of the study are shown in Supplementary Table 2. Analysis of participants' experiences about vaccination led to the emergence of eight main categories at the low point and 10 main categories at the high point. However, four main categories were experienced by participants at both points. Table 1 shows the categories extracted from the participants' experiences during both points.

**Table 1:** Categories explored from the participants' experiences about the vaccination in two low and high peaks.

**Low point**	**High point**	**Common categories**
Weakness in social trust	Disbelief	Disbelief
Uncertainty about the effectiveness of the vaccine	Distrust	Distrust
Lack of trust in the nature of the vaccine	Unawareness and confusion	Unawareness and confusion
Feeling no need to get vaccinated	Forced vaccination	Insufficient facilities
Injustice in vaccine distribution	Doubt	
Disbelief in the existence of the disease	Believing in a high level of immunity	
Challenge in adequate access to vaccines	Changing attitudes	
	Lifestyle	
	Unfulfilled expectations	
	Insufficient facilities	

### Explored categories at the low point

#### Weakness in social trust

The concept of weakness in social trust means that trust in the ability of the authorities to manage the COVID-19 crisis has been weakened due to a sense that preference is given to economic priorities over health priorities, contradictions and lack of coordination, rumors, bitter past experiences, weakness in risk communication. One of the participants said:

“We have heard contradictory ideas about the vaccine. Some people say it is better to be injected, one says an Iranian vaccine will come in 2 months, and one says it will come in 6 months and it would be better to have Iranian vaccine because the rest just have emergency approval, when I consider these words and talk about them I feel that these are not matched, and I do not believe and I do not trust.”

#### Uncertainty about the effectiveness of the vaccine

Uncertainty about the effectiveness of the vaccine was due to reasons such as the lack of sufficient scientific evidence regarding the safety and efficacy of the vaccine in large populations and in the long term, emergency approval of all available vaccines, belief in the non-approval of the vaccine by reputable neutral scientific communities, disagreement among experts regarding the safety and effectiveness of the vaccine, reports of infection after receiving the vaccine, and belief in short immunity duration after vaccination.

One of the health workers said:

“Apart from the possible side effects of the vaccine, it is important how effective the vaccine will be in the long term and what are the long-term side effects of the vaccine? Why do people who get vaccinated get it again around the world? And this is ambiguous yet, so I do not feel good about the vaccine.”

#### Lack of trust in the nature of the vaccine

Participants noted that they did not trust in the vaccine, and this distrust was due to the specific characteristics of the coronavirus, such as the many mutations or reasons such as fear of the negative effects of the vaccine, the unknown long-term effects of the vaccine, hearing news about deaths and complications after the vaccine, not believing that it's possible to “produce a safe and effective vaccine in such a short time,” and uncertainty about the conditions under which the vaccine was made, stored, and transported. One of the participants from the general population said:

“The countries that produced these vaccines are still vaccinating the people, which means that this process is not completed to make sure about the outcome and sometimes I think to myself, what is inside these vaccines? These countries may not report some complications for economic reasons. Normally, it takes about 3–5 years to make a vaccine. How can a safe and effective vaccine be produced in less than a year from the onset of the disease? I think political pressure has led to the emergency approval of vaccines.”

#### Feeling no need to get vaccinated

Some participants stated that they did not feel the need to be vaccinated even if they were given the vaccine. They cited reasons such as the mild nature of the disease, no need for a vaccine after being infected, and high physical strength.

One of the participants from the general population said: “The disease itself ends and goes away. Why should we get vaccinated? Or I have heard that most people get a mild form of the disease. I have a very strong body. The disease does not affect me. I am somehow sure or I will not get sick.”

#### Injustice in vaccine distribution

Some participants noted the lack of confidence in the fair distribution of vaccines, both internationally and nationally, and expressed that, on the one hand, the negative impact of sanctions would prevent Iran from gaining access to the vaccine, and also there is no guarantee of fair distribution of the vaccine in Iran. One of the participants said:

“Sanctions imposed on the Iranian people have limited Iran's access to the vaccines produced and intensified pressure on the people and medical staff in this pandemic. Why should the elderly and medical staff be given priority? “Why not start vaccinating middle-aged people who are active in the community?”.

#### Disbelief in the existence of the disease

Some still do not believe that COVID-19 exists and see it as a conspiracy, while others believe it to be equivalent to the common cold and deny the potential severity of the disease. Some consider that the disease has been created by developed countries to reduce the populations of elderly and vulnerable people, which impose a heavy financial burden on the economy and create a market for vaccines. One of the participants from the general population said:

“COVID-19 is nothing special, it's the same cold and the flu that it used to be... I've been going everywhere for a long time now, I've only had cold symptoms once or twice. Elderly in developed countries need to reduce their population and make money by selling vaccines.”

#### Adequate access to vaccine

One of the main challenges in the vaccine injection process is insufficient supply of the vaccine for all population groups, especially the disabled and house-bound older adults, which was a major concern amongst the participants. Delay in the process of preparation and distribution of vaccines is also one of the main issues. One of the health worker participants said:

“Iran's population is very large and some countries with smaller populations were able to vaccinate very well. Vaccines are not available to everyone, especially the elderly and disabled. Some groups should vaccinate these people at home and we should not wait for them to come. For some people, it is not possible to get out of the house at all.”

#### Explored categories at the high point

##### Compulsory injection

Compulsory injection means that the participants have been vaccinated despite their personal desire to be vaccinated. One of the health worker participants said:

“Several times I was notified about the vaccination of the medical staff, I refused and did not get vaccinated for various reasons, but the hospital announced that those who did not get vaccinated would have their benefits cut off, so I had to get vaccinated.”

##### Doubt

Doubt means ambiguity and mental uncertainty about the treatments for COVID-19 and the effectiveness and safety of the vaccines. One of the participants from the underlying condition group said:

“I had few options regarding the type of vaccine and I was doubtful whether I would receive the vaccine or not. I was doubtful about receiving the vaccine, but because I wanted safety for my family, I injected it.”

##### Believing in a high level of immunity

This category refers to participants' belief in a high level of immunity after receiving both doses of the vaccine. It indicated that participants, after receiving both doses of vaccine, followed fewer health guidelines and were less vulnerable to re-infection and even transmission of the disease. One of the participants from the specific disease stated:

“I've been relieved since I got both doses of the vaccine, I'm less in need of washing my hands or wearing face masks. I injected the vaccine for this reason. I will not get the disease and transmit it to others anymore.”

##### Unfulfilled expectations

Participants assumed that they would no longer develop COVID-19 after receiving two doses of the vaccine. They also believed that after the country was completely vaccinated, the mortality rate would decrease to a greater extent than it has. One of the health worker participants said:

“Vaccination has not been able to reduce a large number of deaths. It is very sad that thousands of people around the world still die every day because of the COVID-19. In countries like the United States, Germany, UK, and Russia, which produced the vaccine themselves and vaccinated it first, thousands of people get infected and die.”

##### Changing attitudes

Some participants changed their attitude toward the vaccine (i.e., became more or less willing to take it) because of their experiences, such as facilitating interaction with members of the community after vaccination and observing the experiences of vaccinated individuals, being influenced by elders and religious leaders, gaining information through the media, economic concerns, and social deprivation due to the disease. One of the participants from the general population stated:

“I wanted to receive the vaccine, but when I saw Bill Gates talk again, I gave up. Bill Gates said that soon there will be a much worse pandemic among vaccinated people than COVID-19.”

##### Lifestyle

In general, this refers to changes in various aspects of participants' daily lives, including the social and psychological dimensions after the COVID-19 vaccine. One of the participants from the elderly group stated: “Because of my job, I have to go out of town twice a week. In the pandemic condition, I either did not go out or if I was going, I was stressed, but fortunately, now that I have been vaccinated, I go twice a week like before.”

#### Concepts common to both high and low points

##### Disbelief

This category indicates a lack of belief in COVID-19. In fact, some participants had no belief in the COVID-19 pandemic and control measures, such as social distancing and the use of face masks. One of the participants from the general population said:

“Now they have sold all the drugs and vaccines as COVID-19 drugs and some of these drugs and vaccines have been completely ineffective at all. COVID-19 has been a trade for them... it was a trade of death, but it does not matter to them. I think that it is not clear whether they inject the vaccine or there is a different story.”

##### Distrust

This category refers to the uncertainty or low trust of the participants at different levels and for various reasons. It indicated the participants' distrust in managers, officials, and experts at the national and international levels, or distrust in conflicting information on the pandemic. One of the participants from the underlying disease group said:

“Each expert says something different about vaccines, you cannot trust that.”

##### Unawareness and confusion

This category means that participants were confused about the presence of COVID-19 as well as whether or not to receive the COVID-19 vaccine. One of the participants from the elderly group said:

“Ordinary people, especially the elderly who can inject now do not have any precise information about the vaccine production process and how it works, so I think it is necessary to give the patient some basic information before the vaccine is given and people do not know what they are injecting.”

##### Insufficient facilities

This category includes concepts that refer to limited and unsuitable capacity to produce, distribute, and manage vaccinations. One of the participants from the general population said:

“For several consecutive days, I went to the center where I received the message, it was very crowded and it was obvious that the number of staff responsible for injections was not enough. There were two people who had to do all injections and they could not do all injections. The number of centers should be increased.”

### Quantitative section

#### Low point phase

The results of this phase of the study have already been published (15) and to compare with the severe phase of the epidemic, some of the most important baseline data from the study are shown in Table 2. In summary, at the low point, the vaccine acceptance was 83.6%, 95% CI: [81.3, 85.9%], and among the participants willing to be vaccinated, 58% preferred international vaccines, 25% Iranian vaccines, and 17% had no preference for vaccine type. Being aged over 60 years [Adjusted Odds Ratio (AOR) = 1.72], 95% CI: [1.01, 2.93], being single (AOR = 0.54), 95% CI: [0.41, 0.91], and moderate drug adherence (AOR = 0.58), 95% CI: [0.4, 0.85], showed statistically significant relationships with willingness to receive the COVID-19 vaccine.

**Table 2:** Baseline characteristics of participants at the low and high points.

**Variable**	**Low point**	**High point**
Age (mean ± SD)	46.5 ± 13.5	41.6 ± 16.2
Male gender	57.80%	51%
**Marital status**		
Married	48.80%	60%
Single	30.40%	28.30%
**Education**		
Illiterate	1.80%	4.60%
Primary and secondary	5.80%	18.70%
High school and diploma	24.10%	35%
Academic	68.20%	41.7
**Self-rated health status**		
Very bad	0.40%	1.20%
Bad	10.40%	4.20%
Moderate	38.80%	22.80%
Good	49.10%	48.90%
Very good	1.30%	22.90%
**Occupation**		
Employed	34.80%	51.70%
Student	21.80%	10.90%
Retired	29.30%	10.10%
Unemployed	2.90%	4.40%
Housewife	11.20%	22.80%
**Chronic disease**		
One chronic disease	18.70%	22.20%
More than one chronic disease	——	7.50%
None	81.30%	70.30%
**Chronic disease in the family**		
One chronic disease	——	28.20%
More than one chronic disease	——	8.10%
None	——	63.70%
**An elderly family member**		
Yes	——	24.60%
**History of COVID-19**		
Yes	15.50%	39.50%
**History of COVID-19 in the family**		
Yes	19.60%	48.30%
**Receiving at least one dose of vaccine**		
**at the time of the study**		
Yes	——	32.40%

#### High point phase

At the peak of the epidemic, data from 1,435 people from 22 urban areas and 437 people from rural areas of Tehran were analyzed. The mean age of participants was 41.0 ± 15.2 years, ranging from 18 to 90 years. Table 2 lists the distribution of baseline variables, self-reported health status, history of chronic disease in the patient and family members, history of COVID-19, and the presence of the elderly among the family members of the studied population. A total of 41.4% of urban participants and 33.2% of rural residents had previously had COVID-19. The history of hospitalization due to COVID-19 was 1 and 6.2% for rural and urban participants, respectively. Also, the incidence of COVID-19 in first-degree family members in urban and rural areas was about 51 and 41%, respectively. At the time of the present study in August 2021, about 79% of rural residents and 64% of urban residents had not received the vaccine. Overall, 20% of participants had received at least one dose of the vaccine, and 75% reported that the most important reason to get vaccinated was to protect themselves and their family members against COVID-19 and related death. “The insistence of family members against one's own desire” (12%), “compulsory vaccination at work and by the employer” (7%), and “others getting vaccinated creating the desire to get vaccinated oneself” (6%) were other causes of vaccination in participants. The frequency of vaccine acceptance (Yes) was 68.5%, 95% CI: [65.8, 71%], and the prevalence of “unsure” and unwillingness to vaccine (No) were 14.7%, 95% CI: [12.9, 16.7%], and 16.8%, 95% CI: [14.8, 19%], respectively. The prevalence of acceptance, unsure, and unwillingness to the vaccine by place of residence, sex, age group, occupation, education, etc. is shown in Table 3. Based on the results obtained from multinomial logistic regression (Table 4), the factors affecting vaccine acceptance were modeled. The odds ratio of unsure to receive the vaccine in rural areas was 0.44 urban areas (*p* = 0.001), while the place of residence was not associated with a unwillingness to the vaccine (*p* = 0.425). Among the participants, students (*p* = 0.011) and housewives (*p* = 0.033) were more likely to receive the vaccine than employees.

**Table 3:** Acceptance, uncertainty, and definite unwillingness to receive the COVID-19 vaccine at the high and low points of the epidemic based on participants' baseline variables.

**Variable**	**COVID-19 vaccine acceptance**			**COVID-19 vaccine acceptance**		
	**prevalence in the high point**			**prevalence in the low point**		
	**Yes** **%(95% CI)**	**NO** **%(95% CI)**	**Not Sure** **%(95% CI)**	**Yes** **%(95% CI)**	**NO** **%(95% CI)**	**Not Sure** **%(95% CI)**
**Age group (year)**						
18–29	72.8 (69.9–77.1)	14.0 (11.6–17.8)	13.2 (11.9–18.9)	81.1 (75.7–85.6)	18.9 (14.4–24.3)	Na
30–49	68.1 (64.0.−71.5)	17.5 (15.7–20.1)	14.4 (12.7–18.3)	84.1 (80.1–87.4)	15.9 (12.6–19.9)	Na
50 and over	64.2 (57.2–70.0)	21.0 (16.2–27.8)	14.8 (11.1–20.8)	84.3 (81.1–87.1)	15.7 (12.9–18.9)	Na
**Gender**						
Male	66.5 (63.1–70.2)	19.7 (17.8–23.9)	13.6 (11.2–17.9)	83.4 (80.0–86.0)	16.6 (14.0–19.5)	Na
Female	70.4 (67.7–74.8)	13.7 (11.5–17.8)	15.8 (13.5–19.2)	83.8 (80.3–86.8)	16.2 (13.2–19.7)	Na
**Marital status**						
Married	68.5 (64.2–71.9)	16.7 (14.2–19.6)	14.8 (12.5–17.6)	85.8 (82.8–88.4)	14.2 (11.6–17.2)	Na
Single	72.0 (67.5–76.1)	15.4 (12.1–19.3)	12.6 (9.6–16.2)	80.5 (76.2–84.3)	19.5 (15.7–23.8)	Na
Divorced	41.8 (28–56)	27.0 (16.3–41.4)	31.2 (19.6–45.7)	81.8 (75.4–86.8)	18.2 (13.2–24.6)	Na
Widow	65.7 (46.1–80.0)	27.5 (14.2–46.6)	6.8 (1.6–24.2)	85.3 (74.7–91.9)	14.7 (8.1–25.3)	Na
**Place of residence**						
City	69.8 (66.2–72.8)	15.4 (12.0–17.1)	17.8 (14.0–19.7)	Na	Na	Na
Village	67.9 (62.5–72.2)	23.8 (19.2–28.9)	10.2 (7.8–13.3)	Na	Na	Na
**Education**						
Illiterate	46.1 (31.1–61.8)	41.0 (26.7–57.1)	12.8 (5.4–27.6)	85.7 (63.1–95.4)	14.2 (4.5–36.8)	Na
Primary and secondary	65.0 (58.3–71.3)	21.0 (15.8–27.0)	14.1 (9.9–19.5)	80.0 (68.9–87.8)	20.0 (12.1–31.1)	Na
High school and diploma	67.8 (63.4–71.4)	18.0 (14.7–21.7)	14.3 (11.4–17.8)	84.5 (79.8–88.2)	15.4 (11.7–20.1)	Na
Academic	71.7 (67.6–75.5)	12.7 (10.0–15.9)	15.6 (12.7–19.1)	83.4 (80.7–85.8)	16.5 (14.1–19.2)	Na
**Self-rated health status**						
Good	71.5 (68.5–74.3)	15.7 (13.5–18.2)	12.9 (10.9–15.2)	82.1 (78.8–85.0)	17.8 (14.9–21.1)	Na
Moderate	60.7 (54.5–66.6)	19.0 (14.7–24.4)	20.2 (15.7–25.7)	84.7 (81.1–87.7)	15.2 (12.2–18.8)	Na
Bad	57.1 (43.9–69.5)	21.4 (12.5–34.2)	21.4 (12.5–34.2)	86.1 (79.0–91.1)	13.8 (8.8–20.9)	Na
**Occupation**						
Employed	67.8 (63.2–70.8)	19.0 (16.2–22.3)	14.4 (12.4–17.9)	82.7 (78.8–86.1)	17.2 (13.8–21.1)	Na
Student	82.1 (75.0–88.7)	8.4 (5.1–14.0)	10.1 (6.5–16.3)	82.3 (77.2–86.5)	17.6 (13.4–22.7)	Na
Retired	72.2 (57.4–84.2)	20.1 (10.0–35.8)	7.8 (2.2–21.5)	85.7 (81.6–89.0)	14.2 (10.9–18.3)	Na
Unemployed	59.8(47.7–71.8)	13.3(6.2–23.2)	28.8(18.8–40.2)	91.6(76.8–97.3)	8.3(2.3–23.1)	Na
Housewife	69.7 (63.1–74.1)	15.1 (11.6–19.5)	17.0 (13.5–21.2)	80.5 (72.9–86.4)	19.4 (13.5–27)	Na
**Chronic disease**						
One chronic disease	54.7 (47.8–61.4)	24.6 (19.2–31.0)	20.7 (15.6–26.8)	84.0 (78.5–88.2)	16.0 (11.7–21.4)	Na
More than one chronic disease	70.7 (57.7–81.1)	13.8 (7.0–25.4)	15.5 (8.2–27.4)	Na	Na	Na
None	71.6 (68.7–74.4)	15.1 (13.0–17.6)	13.2 (11.1–15.6)	83.6 (81.1–85.7)	16.4 (14.2–18.8)	Na
**An elderly family member**						
Yes	67.7 (61.7–73.2)	15.5 (11.6–20.6)	16.7 (12.6–21.9)	Na	Na	Na
No	68.4 (65.5–71.3)	17.2 (15.2–19.7)	14.3 (12.3–16.6)	Na	Na	Na
**History of COVID-19**						
Yes	69.0 (64.7–72.3)	13.7 (11.0–17.0)	17.4 (14.4–21.0)	85.6 (79.8–89.9)	14.4 (10.2–20.1)	Na
No	68.2 (64.7–71.4)	19.1 (16.4–22.1)	12.7 (10.5–15.3)	83.2 (80.7–85.3)	16.8 (14.6–19.2)	Na
**History of COVID-19 in the family**						
Yes	67.2 (63.3–70.9)	15.5 (12.8–18.7)	17.2 (14.4–20.5)	83.5 (78.2–87.7)	16.5 (12.2–21.7)	Na
No	69.5 (65.9–72.9)	18.1 (15.4–21.3)	12.3 (10.0–15.1)	83.5 (81.1–85.8)	16.5 (14.1–18.8	Na

**Table 4:** Multinomial logistic regression of factors affecting doubt and unwillingness to receive the vaccine.

**Variable**	**Doubt**		**Definite**	
			**unwillingness**	
	**Adjusted relative risk ratio**	***p*** **-value**	**Adjusted relative risk ratio**	***p*** **-value**
**Place of residence**				
City	1		1	
Village	0.44 (0.27–0.73)	0.001	1.17 (0.78–1.76)	0.425
**Occupation**				
Employed	1		1	
Student	0.66 (0.32–1.14)	0.124	0.41 (0.20–0.81)	0.011
Retired	0.49 (0.14–1.17)	0.268	0.81 (0.32–2.04)	0.666
Unemployed	1.72 (0.89–3.32)	0.102	0.79 (0.35–1.75)	0.583
Housewife	1.06 (0.70–1.61)	0.768	0.63 (0.41–0.96)	0.033
**Education**				
Academic	1		1	
Illiterate	1.57 (0.51–4.75)	0.268	4.44 (1.91–10.32)	0.001
Primary and	1.14 (0.64–2.00)	0.102	1.41 (0.82–2.40)	0.199
secondary				
High school	1.11 (0.75–1.65)	0.768	1.45 (0.98–2.18)	0.059
and diploma			
**Insurance**				
Yes	1		1	
No	1.5 (1.04–2.24)	0.027	0.92 (0.63–1.35)	0.706
**Underlying disease**				
No	1		1	
One	1.6 (1.04–2.63)	0.031	1.77 (1.15–2.72)	0.009
At least two	1.07 (0.48–2.36)	0.855	0.75 (0.32–1.71)	0.500
**Self-rated health status**				
Good	1		1	
Moderate	1.58 (1.04–2.40)	0.028	1.39 (0.92–2.11)	0.122
Bad	1.54 (0.72–3.25)	0.254	1.54 (0.74–3.12)	0.254

The odds ratio of unwillingness in illiterate people was 4.4 times higher than those with academic education, respectively (*p* = 0.001). Lacking health insurance, having a chronic disease, and self-reported moderate health status were other factors influencing vaccine acceptance.

## Discussion

Our findings showed that about one third of participants had some degree of unwillingness to receive the COVID 19 vaccine. Place of residence, level of education, health insurance, underlying diseases, and moderate health status were factors influencing vaccine acceptance. According to the findings of this study, similar to the low point of the epidemic, in which a small proportion of Iranians were vaccinated, distrust in the effectiveness and safety of vaccines and vaccine companies were the main reasons for doubt and unwillingness to receive the COVID-19 vaccine. The quantitative and qualitative studies showed that, in addition to lack of trust, other factors, including confusion regarding the vaccination process, the possibility of commercialization of the disease and vaccine, lack of proper information from officials, and compulsory vaccination due to organizational considerations were factors contributing to vaccine hesitancy.

### Comparison of low and high points

The city of Tehran, as the political and economic capital of Iran, has a wide variety of people with different social, economic, and ethnic backgrounds; therefore, the results of our study can cautiously be taken to indicate the status of vaccine acceptance in Iran. Our findings showed that at the high point, 68% of participants were willing to receive the vaccine, and slightly less than a third were either unwilling to receive the vaccine, or certainly unwilling to be vaccinated with any type of COVID-19 vaccine. The prevalence of vaccine acceptance has been reported in population-based studies in various countries, such as Bangladesh (74%) (16), USA (67%) (17), Jordan (72%) (18), Saudi Arabia (64%) (19) and Italy (54%) (20). In addition to the methodological differences in the running of the studies that will be discussed in the next section, the differences in the social and cultural structures of the countries, the availability of safe vaccines, as well as the incidence of new cases and deaths are among the most important factors affecting the prevalence of vaccine acceptance. Therefore, the comparison of countries will be limited, and for a more correct comparison of the COVID-19 vaccine acceptance status, consecutive studies should be conducted in the epidemic period. In the first phase of our study, at the low point of the epidemic, about 84% of participants were willing to receive the vaccine (14) and, compared to the results of other studies, we were among the countries with the highest rates of vaccine acceptance. It is noteworthy that at the low point, due to sanctions, insufficient access to international vaccines, and the commencing of vaccination in some countries with an expedited approval process, the willingness to get vaccinated was high among Iranians. However, at the high point, despite a large number of hospitalizations and deaths due to the occurrence of the fifth wave, as well as the ease of access to the vaccine, the willingness of people to receive the vaccine had decreased. The main reasons for this decrease can be explained based on the results of the two qualitative studies: the continuing of the epidemic with multiple waves in countries that have access to high-quality vaccines, the spread of unofficial and contradictory news about the new cases, and the mortality of people vaccinated with COVID-19 has led to a decrease in trust in the effectiveness of the COVID-19 vaccine and strengthened the point of view that the COVID-19 vaccine is commercial, thus decreasing willingness to get vaccinated. In other words, the epidemic wavescan have an adverse effect on the speed of vaccination and over time, the positive psychological effects of vaccination will decrease. Inadequate distribution of vaccines and lack of transparency and consistency about vaccination status are other reasons that may justify a decrease in the willingness to receive vaccines during the high point of the epidemic. Therefore, in communities with a low level of trust in vaccine efficacy, a lack of proper vaccination planning can also negatively affect people who definitely want to be vaccinated or are doubtful about being vaccinated. Our results showed that 16.7% of participants were unsure about receiving the vaccine and half of participants reported a “low” or “very low” level of coordination of the information about the COVID-19 vaccine provided by the officials. This contradiction and its negative effects can be greater on people with a low level of trust in the vaccine, and, as a result, 17% of doubtful people became completely unwilling to get vaccinated. Conflict and the presence of experts in the media with different specialties and attitudes toward the vaccine on the one hand and letting demonstrations in the city can increase the unwillingness and uncertainty about receiving the vaccine. In addition, rising morbidity and mortality rates in developed and vaccine-producing countries with widespread and rapid vaccinations compared to other countries, such as the United States, Russia, the United Kingdom, and Germany, have raised doubts about the vaccine's effectiveness.

According to the results, at the time of the study, up to 50% of participants had a history of COVID-19 in themselves or family members, and a sense of safety and no need to receive the vaccine can play an important role in reducing willingness to get vaccinated. Similarly, regarding booster dose injection, previous infection and having previously received two doses of vaccine can greatly reduce people's motivation to ensure humoral and cellular immunity, and in the qualitative study, we showed that believing in a high level of immunity and lifestyle changes can be an influential factor in the vaccination process. In general, it seems that the longer the duration of an epidemic and especially the longer the trend is affected by the emergence of new variants, the greater the spread of doubt in vaccine effectiveness in the community through social media and vaccine opponents, thus making it more difficult to persuade people to get the vaccine or a booster dose. In a country like Iran, where national and international vaccines are currently available and the new Omicron strain is increasing with delay in Iran compared to European and American countries, as of writing this, about 70% of eligible people have received two doses of the vaccine and about 30% have received three doses of the vaccine, illustrating that the complexity of human behavior in long-running epidemics requires mixed methods studies over a period of time. Therefore, the change in people's behavior and the causes of this change should be monitored.

### Methodological issues

Although various studies have reported vaccine acceptance rates in different populations (21), comparisons of quantitative results have been limited due to the use of questions with different categories in response options. Some studies, such as our first phase study reported willingness to receive the vaccine using yes/no options, and may therefore overestimate the rate of vaccine acceptance due to incompatibility with the proposed structure of the SAGE Working Group on Vaccine Hesitancy (22). According to the results of cross-sectional studies based on the general population, in which only yes/no options were considered, the vaccine acceptance rate in Bangladesh was 79% (23), in Lebanon was 64% (24), in South Korea was 53% (25), and in Brazil was 81.4% (26); in our first phase vaccine acceptance was about 84% (14). Because the willingness to receive the vaccine is not a binary decision and a range of emotions and factors can be involved, in other studies, such as our study in Phase II, the response to the willingness to receive the vaccine was reported as “acceptance,” “definite unwillingness” and “not sure.” In these studies, “acceptance” and “uncertainty” in the general population over the age of 18 were, respectively, 65 and 27% (19) in Saudi Arabia, 85 and 9.4% in Australia (27), and 57.6 and 31.5% in the United States (28). Other studies have examined vaccine acceptance using a 5-point or even 6-point Likert scale from “I will definitely get vaccinated” to “I definitely will not get vaccinated”; depending on the conditions in each country, the answers can tend to one of the two ends of the spectrum (29, 30). Therefore, it is recommended that researchers refrain from examining the acceptance of the vaccine in binary form, and while estimating the amount of uncertainty regarding vaccination, the course of change in the tendency of people to receive or not receive the vaccine in uncertain people be determined and the factors affecting this change using longitudinal studies be monitored.

### Predictive factors

Review studies have shown (9, 21, 31) some important underlying factors, such as the age and sex of individuals and their impact on vaccine hesitancy are not consistent and because of the social roles of men and women in the target population, social trust, and health literacy status, in some studies, women are more reluctant to receive the vaccine (32–34); in other studies, men as a group have been reported to be more likely to be vaccinated (35–37); and in still other studies, such as our two study phases, the relationship between gender and vaccination doubts was not significant (14, 28, 38). In some studies, regardless of gender, a low level of education has been an important risk factor for vaccination hesitancy (5, 39, 40) and as shown in our final model, illiterate people were about 4.5 times less likely than educated people to get the COVID-19 vaccine. In accordance with our findings, Abedin et al. showed in a large survey in eight districts of Bangladesh that people who had schooling of more than 12 years were 65% less likely of vaccine hesitancy compared to people without formal-education (16). The significant role of the Internet and the spread of information in this context and its irreplaceable impact on increasing the perception of danger as well as the deprivation of illiterate people to take advantage of the Internet, no high socio-economic level, and no priority for COVID-19 in their everyday life can be one of the most important mediators of unwillingness to receive the vaccine in illiterate people. A qualitative study on illiterate people and an in-depth analysis of the reasons for the unwillingness of this group of society is recommended in future studies.

Underlying diseases, such as diabetes, hypertension, cardiovascular disease, cancer, disability, etc. have been another factor in the rejection of the vaccine. Our findings showed that in people with a chronic illness who take medications, definite unwillingness to receive the vaccine is 77% higher, and they are 60% more reluctant to receive the vaccine than those who are in good health. Similar to our study, cross-sectional population-based studies in Hong Kong (41), Bangladesh (16), Turkey (42), and France (43) also reported that in people with underlying diseases, uncertainty about receiving the vaccine due to fear of unknown side effects and drug interactions in these patients is less than the general and healthy population. In this regard, cases who did not report good health status using the standard self-rated health status question and who felt they had moderate physical health were more doubtful about receiving the vaccine. In some clinical trial studies, COVID-19 vaccines have been considered as an exclusion criterion for underlying diseases, such as cancer, stroke, immunosuppressive drugs, etc. (44). Therefore, the generalization of the effectiveness and occurrence of side effects of these vaccines to people with underlying conditions is limited. On the other hand, due to weaker immune system and the use of immunosuppressive drugs in some of these people, they are at much greater risk of severe forms of COVID-19, and as a result vaccination of this group is of great importance in terms of public health and reducing the infection and mortality in society. Therefore, the use of vaccines with scientific approval for patients with underlying diseases and increasing patients' awareness and trust in these vaccines should be considered by medical staff and physicians treating these patients.

### Strengths and limitations

Some of the strengths of the present study are conducting a two-phase study because, in some previous review studies on vaccine acceptance, the lack of multi-phase studies has been mentioned as a limitation of studies, and has also been noted that due to the changeable nature of vaccine acceptance, it is not possible to study the willingness to get vaccinated against COVID-19 in a population using one study. Junjie et al. in their scoping review reported a lack of qualitative studies on the acceptance of the COVID-19 vaccine as one of the limitations (15); therefore, we conducted two qualitative studies to identify and understand unwillingness to receive the vaccine and the vaccination process. The study was designed to have appropriate sample size, cluster sampling, and data collection with face-to-face interviews; however, many studies have been conducted online using snowball sampling, and the sample may not represent the entire population due to the limitations of this method. In addition to the strengths, this study had some limitations, including differences in some items of the two-phase questionnaire, which limits ability to compare the two time points. Although the research team tried to minimize the difference between the samples of two study phase by using representative samples from the city of Tehran, another limitation of the present study is the difference in the participants in the two studies, which limits the comparison of vaccine acceptance over time. Furthermore, measurement error and random error were other methodological limitations affecting the results of the two studies.

## Conclusion

Unexpectedly, the high peak of the epidemic compared to the low peak in Iran did not increase vaccine acceptance, and some obstacles, such as increasing distrust in vaccine efficacy due to multiple peaks in different countries with international vaccines, as well as the influence of the media, opponents of the vaccine, and the lack of proper communication, has increased vaccine hesitancy. Illiterate people with underlying conditions who are not in good health are more unwilling to receive the vaccine, and rural communities are less unwilling to receive the vaccine than urban residents. At the time of this study, we are facing an increase in the number of cases with the Omicron strain in Iran, and it is expected that considering the results of these two mixed methods studies, health policymakers in Iran will pay more attention to the risk communications, swaying public opinion, and increasing public trust, as these are key factors for vaccine acceptance, willingness to get booster doses, and. Further investigation into factors influencing vaccine hesitancy over the long term is recommended.

## Data availability statement

The raw data supporting the conclusions of this article will be made available by the authors, without undue reservation.

## Ethics statement

The study was conducted according to the guidelines of the Declaration of Helsinki and was approved by the Research Ethics Committee of the University of Social Welfare and Rehabilitation Sciences (code: IR.USWR.REC.1400.222). Informed consent was obtained from all subjects involved in the study. The patients/participants provided their written informed consent to participate in this study.

## Author contributions

HK, MH-F, and MS contributed to the conception and design of the study. MS, MP, and MH-F wrote the first draft of the manuscript. MR and MK wrote sections of the manuscript. SA, EG, and AH managed the database. MS, MP, MH-F, and AH performed the statistical analyses. All authors contributed to manuscript revision, read, and approved the submitted version.
