# Supplementary material for: Comparison of vaccine hesitancy during the low and high points of COVID-19 in a population under international sanctions: A longitudinal mixed-methods study in Iran
Source: Front Public Health. 2023 Jan 5;10:958899. doi: 10.3389/fpubh.2022.958899 (PMC9850560; doi:10.3389/fpubh.2022.958899)
Supplement: Supplementary file 1 [file Table_1.docx]

**Supplementary**

**Supplementary Table 1**: Classification of data collected in the second phase of the study on COVID-19 vaccine acceptance in Tehran and its villages

| Row | Categories | Variables |
| --- | --- | --- |
| 1 | Demographic and baseline characteristics | Age, sex, marital status, level of education, chronic disease in the person and his family members, the existence of elderly in family members, place of residence, a standard question on self-rated health to assess participant’s health status |
|  | COVID-19 infection and death status | History of getting COVID-19 before starting the present study,  Family history of COVID-19-related death in family members |
|  | Vaccine history and willingness to receive the vaccine | History of receiving COVID-19 vaccine, reasons for receiving the vaccine, willingness to receive the vaccine in participants who have not been vaccinated by the time of the study, the tendency to national or international type of vaccine, the most important reasons for reluctance or hesitation to receive the vaccine and change the tendency to inject the vaccine in low and high peaks of the epidemic |
|  | Receiving information about the vaccine and satisfaction with the process of vaccination in Iran | Sources for receiving information about the vaccine, satisfaction with the provision of the vaccination service, informing the authorities about the vaccination process |
|  | Risk perception | Consequences of receiving and not receiving the vaccine |

**Supplementary Table 2: Characteristics of the participants in the qualitative part of the study**

| High peak(n) | Low peak(n) |  |
| --- | --- | --- |
| 42  12  8  12  10 | 45  11  9  11  14 | Participants(Total)  General population  Healthcare provider  Patients with certain diseases  Elderly |
| 19  23 | 24  21 | Gender  Female  Male |
| 7  4  14  1  8  6  2 | 6  10  13  1  13  2  - | Education  Medical doctor  Master  Bachelor  Associate  Diploma  Elementary  Illiterate |
| 24-95, Mean:42 |  | Age(year) |

**Supplementary Table 3: multinomial logistic regression of factors influencing doubt and unwillingness to receive the vaccine**

|  | Relative risk ratio | | P-Value | Relative risk ratio | P-Value |
| --- | --- | --- | --- | --- | --- |
| Variable | **Doubt** |  | | **Unwillingness** |  |
| Place of residence |  | | | | |
| Urban | 1 |  | | 1 |  |
| Rural | 0.59 | 0.011 | | 1.6 | 0.003 |
| Age group |  | | | | |
| 18-29 | 1 |  | |  |  |
| 30-49 | 1.15 | 0.430 | | 1.35 | 0.096 |
| 50 and over | 1.21 | 0.410 | | 1.75 | 0.012 |
| Gender |  | | | | |
| Female | 1 |  | |  |  |
| Male | 0.91 |  | | 1.5 | 0.008 |
| Education |  | | | | |
| Academic | 1 |  | | 1 |  |
| Illiterate | 1.27 | 0.643 | | 5.02 | 0.000 |
| Primary and secondary | 0.99 | 0.237 | | 1.8 | 0.007 |
| High school and diploma | 0.96 | 0.176 | | 1.4 | 0.028 |
| Occupation |  | | | | |
| Employed | 1 |  | | 1 |  |
| Student | 0.56 | 0.065 | | 0.34 | 0.001 |
| Retired | 0.49 | 0.245 | | 0.96 | 0.924 |
| Unemployed | 2.25 | 0.008 | | 0.73 | 0.440 |
| Housewife | 1.15 | 0.446 | | 0.75 | 0.143 |
| Insurance |  | | | | |
| Yes | 1 |  | |  |  |
| No | 1.41 | 0.043 | | 1.35 | 0.104 |
| Chronic disease |  |  | |  |  |
| No | 1 |  | |  |  |
| One | 2.04 | 0.001 | | 2.1 | 0.001 |
| At least two | 1.18 | 0.625 | | 0.92 | 0.840 |
| History of COVID-19 |  | | | | |
| Yes | 1 |  | | 1 |  |
| No | 0.73 | 0.068 | | 1.41 | 0.035 |

| History of COVID-19 in family |  |  |  |  |
| --- | --- | --- | --- | --- |
| Yes | 1 |  | 1 |  |
| No | 0.69 | 0.025 | 1.12 | 0.452 |
| Self-rated health status |  | | | |
| Good | 1 |  | 1 |  |
| Moderate | 1.85 | 0.001 | 1.41 | 0.059 |
| Bad | 2.08 | 0.038 | 1.70 | 0.126 |
| An elderly in the family |  | | | |
| Yes | 1 |  | 1 |  |
| No | 0.84 | 0.394 | 1.09 | 0.639 |
| Level of development of the place of residence |  | | | |
| Developed | 1 |  | 1 |  |
| Relatively developed | 1.19 | 0.634 | 0.75 | 0.412 |
| Moderate development | 0.89 | 0.712 | 0.37 | 0.001 |
| Less developed | 1.39 | 0.304 | 0.74 | 0.320 |
| Undeveloped | 1.41 | 0.306 | 0.69 | 0.276 |
